# Investigation of image-guided in vivo irradiation on voiding patterns and bladder contractility in female mice

**DOI:** 10.1038/s41598-025-30020-6

**Published:** 2025-12-13

**Authors:** Sarah McDowell, Conor Breen, Niamh McKerr, Kirtiman Srivastava, Daniel Crummey, Mihaela Ghita-Pettigrew, Karl T. Butterworth, Joe M. O’Sullivan, Kevin M. Prise, Karen D. McCloskey

**Affiliations:** 1 Johnston Cancer Research Centre, School of Medicine, Dentistry and Biomedical Sciences, Queen’s University Belfast, 97 Lisburn Road, Belfast, BT9 7AE UK; 2 Northern Ireland Cancer Centre, Belfast City Hospital, 97 Lisburn Road, Belfast, BT9 7AE UK

**Keywords:** Bladder, Radiotherapy, Voiding, Neurogenic contractions, Physiology, Oncology, Urology

## Abstract

Radiotherapy for pelvic malignancies impacts the bladder causing urinary symptoms in many patients. Preclinical models using image-guided irradiation enable investigation of the physiology underlying radiation-response in the bladder. We aimed to characterise voiding patterns and bladder contractility following image-guided irradiation of the mouse bladder. Bladders of adult, female mice were irradiated (IRR) with a single fraction (20 Gy) under cone beam computed tomography. Pre- and post-IRR, void spot assays of urination patterns were performed. Contractility of bladder strips was quantified with myography. At 2-weeks, 1-month and 2-months post-IRR, around 50% of mice exhibited increased void locations. At 2-weeks, 50% had increased void spot counts; moreover, the majority exhibited smaller mean and smaller maximum void spot volumes. In bladder strips, neurogenic-contractions were smaller at 2-weeks, independent of their voiding patterns. Some recovery of contraction-amplitude was noted up to 8-months. At 2-weeks, depolarization-contractions and carbachol-contractions were similar in non-IRR and IRR strips; however, ATP-contractions were smaller. Neurogenic-contractions, carbachol-contractions and ATP-contractions had similar sensitivity to the SK channel blocker, apamin. Neurogenic contractions had similar sensitivity to paxilline (BK) and TEA (pan-K^+^) in non-IRR and IRR tissue. Targeted bladder irradiation resulted in altered urination patterns in the acute post-IRR phase, coincident with reduced neurogenic-contractions which might limit voiding. The ability of the bladder to contract per se and the contribution of K^+^-channels to contractility was minimally impacted by irradiation. Whilst the underlying mechanisms of the observations require further investigation, the study demonstrates the application of image-guided irradiation for quantifying radiation-induced changes in mouse bladder.

**Supplementary Information:**

The online version contains supplementary material available at 10.1038/s41598-025-30020-6.

## Introduction

Radiotherapy for pelvic malignancies, although effective, may cause complications in normal bladder^1^. Whilst technological advances enable more precise delivery of radiation to tumours, neighbouring tissues including the bladder can exhibit radiation-induced dysfunction. Many patients undergoing pelvic radiotherapy experience urinary symptoms that develop acutely during treatment and up to 90 days post-treatment, or manifest as late/chronic effects in subsequent months/years following a latency period. Acutely, radiation cystitis is characterised by haematuria, bladder irritation, pain, frequency, nocturia and urgency^2–4^. Resolution of acute symptoms is common; however, late effects are irreversible and may include incomplete emptying, nocturia, frequency, urgency or severe haemorrhagic cystitis—attributed to ischaemia and tissue fibrosis.

The incidence of radiation-induced bladder dysfunction varies across cancer types and mode/dose of therapy. Browne et al. reported an incidence range of radiation cystitis as 23%-80%, and 5%-8% for severe haemorrhagic cystitis^5^; moreover, others reported incidence of 3%-39%^6^. In female pelvic radiotherapy, the incidence of acute effects is 28%-45% (Grade 2 toxicity) whereas late effects (Grade 3 toxicity) occurring 3 or 20 years post-treatment have reported incidences of 7.7% and 14.4% respectively^4^. In prostate cancer, the CHHiP trial reported the estimated cumulative 5-year incidence of ≥ Grade 2 bladder-dysfunction as 9.1%-11% (around 30% in weeks 1–8)^7^. Whether attributed to acute or late effects, urinary dysfunction even if mild, impacts health and quality-of-life.

Pre-clinical models enable investigation of the underpinning pathophysiology. Early murine studies utilised irradiation of the pelvis, or, targeted the bladder using lead shielding^8–10^. Further refinement using an external abdominal band to physically reposition the intestines away from the radiation field, reduced gastrointestinal toxicity and improved animal welfare^11^. These studies successfully demonstrated reduced bladder capacity of < 50% of pre-irradiated levels. An alternative model opened the abdomen and directly irradiated the bladder through a surgical window^12^ before repositioning*.* While this methodology does not replicate the clinical setting, it induced significant radiation-bladder toxicity and enabled investigation of of human relaxin-2 to ameliorate urinary symptoms^12^.

Implementation of cone beam computed tomography (CBCT) image-guidance into pre-clinical radiation systems is a significant refinement of conventional in vivo radiobiology techniques that enables new lines of laboratory research^13–15^. Zwaans et al. (2016) used an image-guided approach to deliver a single-fraction of 20 Gy to the mouse bladder, calculated to mimic clinical dose regimens e.g. 2 Gy × 37 fractions used in prostate cancer treatment^16^. This targeted bladder irradiation evoked a radiation-bladder phenotype i.e. altered micturition, early inflammation, late fibrosis and bladder wall remodelling over a 5-month period^16^. They later reported that 40 Gy also caused altered micturition in female mice acutely (4–8 weeks), correlated with urothelial thinning, loss of urothelial adhesion and tight junction proteins and decreased uroplakinIII expression, all which resolved 12-weeks post-irradiation^17^. In contrast, 30 Gy targeted to bladders of male mice did not impact micturition acutely (4 weeks), but increased voiding frequency and decreased void volumes after 14-weeks^18^.

Altered bladder function post-irradiation likely represents multiple mechanisms including those related to bladder contraction. We and others have shown that e*x vivo* irradiation of guinea-pig bladder or in vivo irradiation of rat bladder, using a single 20 Gy fraction resulted in reduced amplitude of neurogenic contractions in myography studies^19,20^.

The present study builds on this body of knowledge and aimed to investigate the impact of targeted irradiation at acute and later timepoints on (1) mouse bladder voiding patterns and (2) several key parameters of bladder contractility.

## Materials and methods

### Animals

C57Bl/6 adult female mice (8-weeks, Charles River, United Kingdom) were maintained in a core facility with ad libitum access to food and water. The study focussed on female mice as it incorporated evaluation of urination patterns which could be complicated by male mouse territory-marking^16^. This age group was selected to enable the impact of radiation on the bladder to be assessed over the time course (up to 8-months) without confounding factors of ageing. Animal health and wellbeing was monitored by core facility staff and two researchers. Procedures were carried out under project licence PPL2826 (Department of Health, Northern Ireland), with approval of the Animal Welfare Ethical Research Board at Queen’s University Belfast, in accordance with relevant guidelines and regulations, and are reported in accordance with ARRIVE guidelines^21^.

### Irradiation

Imaging and irradiation (IRR) were performed using 220kVp X-rays under CBCT image-guidance using a Small Animal Radiation Research Platform (SARRP, Xstrahl Life Sciences, Camberley, UK), calibrated using the Institute of Physics and Engineering in Medicine and Biology code of practice^22^. Mice were randomised to treatment groups: non-irradiated (non-IRR) or irradiated (IRR). Animals received random, unique identifier numbers, were assigned randomly to numbered cages (4 mice/cage) and ear-punched according to an in-house naming convention. Mice were weighed prior to irradiation, weekly thereafter in the acute phase and prior to sacrifice for myography. A total of 62 mice are within the present study; the Void Spot Assay (VSA) contains data from 32 mice with pre-IRR and post-IRR readouts across a range of timepoints (the number of mice at each timepoint is given in the results), and the myography experiments contain data from 16 non-IRR mice, and 35 post-IRR mice at various timepoints (details given in the results).

Mice were anaesthetised with 3% isoflurane for induction and 1.5% for maintenance throughout imaging and irradiation. They were placed supine on a mouse bed to aid bladder targeting. CBCT scans were acquired (60 kV, 0.5 mm Al filtration) pre-IRR for target delineation then 20 Gy was delivered as a single fraction using an anterior–posterior beam geometry configuration to the whole bladder (10 × 10 mm field size, dose rate 3.12 ± 1.8 Gy/min). Post-IRR, mice recovered from anaesthesia in a heated chamber and were returned to cages when fully conscious and moving freely.

### Void spot assay

**Fig. 1:**
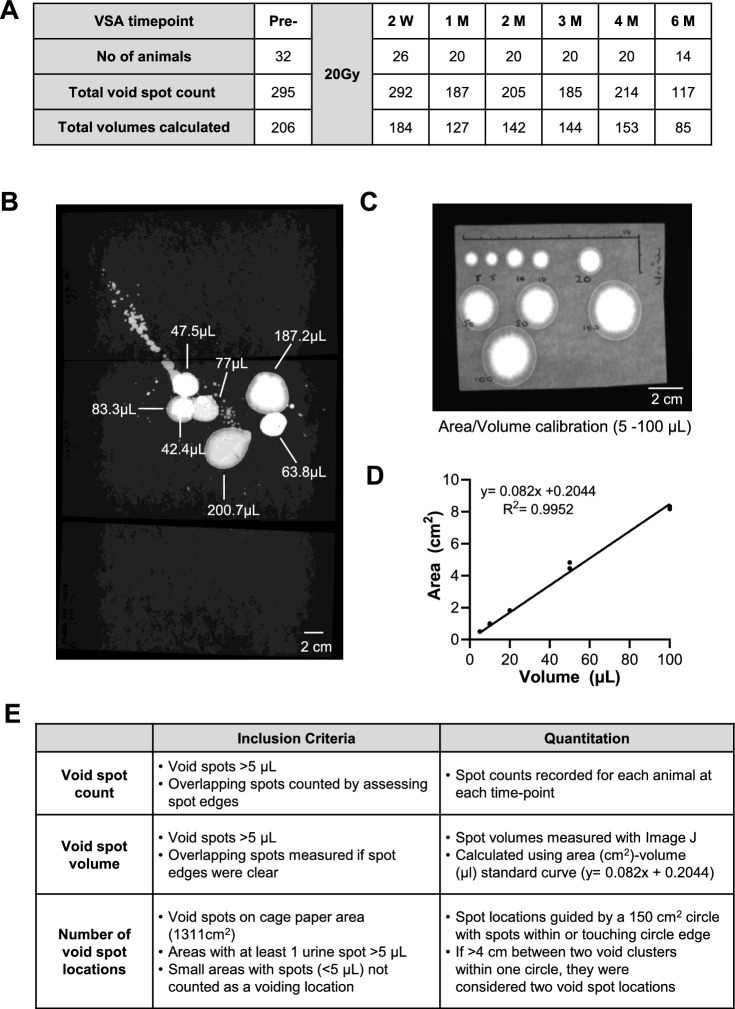
Void spot analysis. **A** Summary of Void Spot Assay (VSA) protocols. Mice were housed in metabolic cages containing absorbent paper during the active period (16:00 to 08:00 the following day). Some mice were randomised to receive 20 Gy via a CBCT image-guided platform (SARRP) and were re-evaluated across subsequent time-points shown. Total void spots included in analysis and the number of volumes that could be calculated from the void spots are indicated for each time-point. **B** The filter paper was cut into 3 parts to fit within a UV imager and realigned digitally for analysis. The 3 sections are visible in the composite image. Voids spots could be visualised, some of which were overlapping. **C** A void spot area to volume calibration was carried out by spotting known volumes (5, 10, 20, 50 and 100 μL) of human urine (available from a clinical study) to filter paper in duplicate and then imaging under UV light. A black background was used to highlight the edges of the paper and a scale (10 cm) was drawn for reference. **D** Spot areas from panel C were measured in Fiji (Image J) and then plotted against volume. Data was fitted with a y = mx + c function and the R^2^ calculated. This was subsequently used to calculate the volume of void spots. **E** VSA inclusion criteria and parameters used for measurements of void spot count, void spot volume and the number of void spot locations in the cage.

Mice were placed in individual rodent metabolic cages with ad libitum access to food and water for Void Spot Assay (VSA) of urination patterns pre-IRR and post-IRR timepoints; 2-weeks (2W-IRR), 1-month (1M-IRR), 2-months (2M-IRR), 3-months (3M-IRR), 4-months (4M-IRR) and 6-months (6M-IRR) (Fig. 1A). Mice acclimatised to the environment for 1 h before filter paper was placed on the cage base. Voiding patterns were assessed between 16:00 and 08:00 the following day, a 16-h period when mice are awake and active^16^. The paper was later imaged under ultraviolet light (Syngene G:Box Chemi XX6 system). The paper (approx. 28.5 cm × 46 cm) was cut into three labelled sections, imaged and the images aligned digitally (e.g. composite image, Fig. 1B). Calibration comprised spotting known volumes of human urine (available from a clinical study; 5, 10, 20, 50 and 100 μL) on filter paper, imaging (Fig. 1C), calculating spot areas in Image J (National Institutes of Health, USA^23^) and creating a standard curve, fitted by a linear regression function in GraphPad Prism (v10.4.1) (Fig. 1D).

Inclusion criteria and quantification are given in Fig. 1E as per published recommendations ^24^. Wegner et al. recommended inclusion of micro-spots and considered that they are unlikely to be explained by mouse tail dragging or depositing of micro-urine spots attached to the animal’s fur ^25^. However, automated analysis of micro-spots was not possible here due to the image file properties that were incompatible with the automated platform. A threshold was used where void spots < 5 µL were excluded from manual analysis (Fig. 1B). It was possible to visualise and disaggregate the majority of overlapping void spots so that void spots could be counted and measured. Where overlap interpretation was difficult, an estimation of the number of void spots was made by assessing spot edges.

**Fig. 2:**
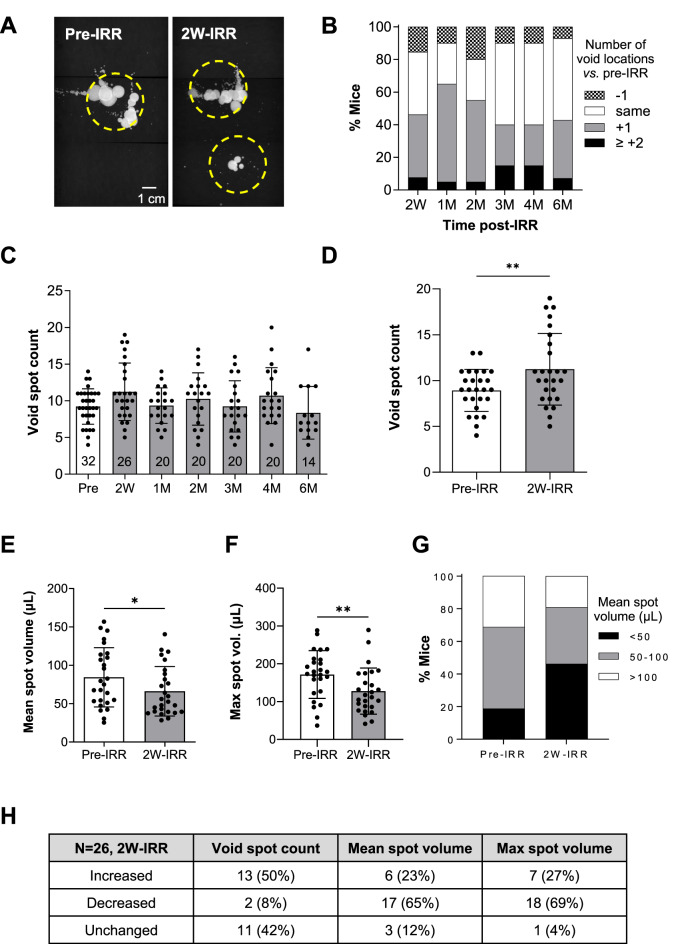
Irradiation affected voiding location, void spot volume and void spot counts. **A** Example of void spot patterns from a mouse pre-irradiation (IRR) and 2 weeks post-IRR. Each image is a digital composite of 3 images as described in Fig. 1B. The yellow dashed circles show voiding locations as per the criteria summarized in Fig. 1E. **B** Summary data showing the percentage of mice with increased, decreased or unchanged number of voiding locations after irradiation at each timepoint. Up to 2M, a higher percentage of mice had more voiding locations than those which had no change, whereas from 3M, a higher percentage of mice showed no change from pre-IRR patterns. **C** Void spot count at each timepoint for all animals (N indicated within the bars) is presented as mean (SD). **D** Void spot count for the 2W-IRR cohort was increased compared with matched pre-IRR counts (N = 26). Data are presented as mean (SD) and data sets were compared with paired t-tests, **denotes *P* < 0.01. **E** Mean spot volume for the 2W-IRR cohort was decreased compared with matched pre-IRR data (N = 26). Data are presented as mean (SD) and data sets were compared with paired t-tests, *denotes *P* < 0.05. **F** Maximum spot volume for the 2W-IRR cohort was decreased compared with matched pre-IRR data (N = 26). Data are presented as mean (SD) and data sets were compared with paired t-tests, **denotes *P* < 0.01. **G** Void spot volumes were binned into small (< 50 μL), medium (50–100 μL) and large (> 100 μL) categories for pre-IRR and 2W-IRR data. A higher percentage of mice had small volume void spots 2W-IRR, and a smaller percentage of mice had large volume void spots 2W-IRR. **H** Summary data for the 2W-IRR cohort showing numbers (N) and percentage of animals with increased, decreased or unchanged readouts of void spot count, mean spot volume or maximal spot volume.

Spot volumes were calculated from the calibration curve, y = 0.082x + 0.2044 (Fig. 1C). The area of any overlapping void spots was measured if clear spot edges were present. Void spot locations were guided by a 150 cm^2^ circle-guide and spots that were within, or touching the circle were accepted for analysis (Fig. 2A). If > 4 cm existed between 2 spots within the same circle area, they were considered as two separate void spot locations. Void spot locations on the filter paper area (1311 cm^2^) were assessed as follows: (1) a location was defined as having at least 1 spot > 5 µL, and (2) regions of micro-spots were not counted as a void location. Summary data are presented as mean ± standard deviation (SD) and granular points included where relevant (GraphPad Prism, v10.4.1). Data were compared using paired t-tests or ANOVA with *P* < 0.05 considered as significant. In vivo transurethral cystometry recordings were carried out to evaluate the effect of irradiation on bladder pressure–volume relationships ^11^; however, a technical artifact led to the recordings being deemed inconsistent.

### In vitro* myography*

Mice were sacrificed by CO_2_ inhalation according to Schedule 1 of the Animal (Scientific Procedures) Act (UK, 1986). Mean animal weights ± SD (g) for non-IRR, 2W-IRR, 4 M-IRR, 6 M-IRR, non-IRR 8 M and 8 M-IRR were 22.4 ± 2.67 (10), 20.4 ± 1.52 (18), 22.8 ± 1.32 (6), 25 ± 1.91 (6), 27.2 ± 2.41 (6) and 28.8 ± 2.84 (11) respectively with N indicated in parenthesis. After sacrifice an incision was made in the lower abdomen and the lower urinary tract removed. The bladder was dissected free of surrounding fat and connective tissue, weighed, opened longitudinally and 4 longitudinal strips (2 × 2 × 6 mm) were cut for in vitro myography as described previously (McDonnell et al., 2018). Mean bladder weights ± SD (g) for non-IRR, 2W-IRR, 4 M-IRR, 6 M-IRR, non-IRR 8 M and 8 M-IRR were 0.017 ± 0.003 (10), 0.018 ± 0.003 (18), 0.024 ± 0.004 (6), 0.019 ± 0.002 (6) and 0.019 ± 0.002 (6) and 0.019 ± 0.003 (10) respectively with N indicated in parenthesis. Bladder strips were used as full-thickness and the mucosal layer was not removed. Strips equilibrated after an initial tension was applied (10 mN, 1 h) and typically relaxed and were readjusted to 10 mN up to three times if necessary during the first 10 min. Tissues were perfused with Krebs’ solution (2 mL/min) aerated with a 95% O_2_/ 5% CO_2_ mixture at 35–37 °C. Data was acquired with Chart software (v4.0.1, University of Strathclyde, UK).

Neurogenic-contractions were generated via electrical field stimulation (EFS) 0.3 ms pulse width, 40 V, 10 s duration across a frequency range (0.5, 1, 2, 4, 8, 16, 32 Hz). Depolarization-contractions were induced by 60 mM K^+^ (high-K^+^) Krebs’. Cholinergic and purinergic post-synaptic receptor-contractions were generated by perfusion of carbachol or addition of ATP directly to the organ baths. Peak contraction amplitude was measured relative to baseline in Chart, or with Clampfit (pClamp v10.3) following file conversion to .abf using WinEDR (v3.5.6, University of Strathclyde, UK). Area under curve (AUC) analysis of carbachol responses was performed in Clampfit in which the first three minutes of contractile responses were measured. Data was recorded in an Excel workbook (Microsoft Office) and statistical analysis performed using GraphPad Prism (v10.4.1). Data are presented as bar charts (mean ± standard error of the mean (SEM)). In supplementary data, results graphs are presented as mean ± SD along with granular datapoints. Throughout, ‘N’ and ‘n’ refer to the number of animals and number of bladder strips respectively. Statistical tests are indicated in figure legends.

Krebs’ comprised (mM): NaCl (120); KCl (5.9); NaHCO_3_ (25); glucose (5.5); NaH_2_PO_4_ (1.2); MgCl_2_ (1.2); CaCl_2_ (2.5). High K^+^ (60 mM) Krebs’ had substitutions in NaCl and KCl. Components were obtained from Sigma-Aldrich. TEA (tetraethylammonium chloride, T2265, Sigma-Aldrich) was dissolved in Krebs’ to its final concentration. Paxilline (P2928, Sigma-Aldrich) was dissolved in DMSO and diluted to final concentration in Krebs’. Apamin (A9459, Sigma-Aldrich), Carbachol (2810, Tocris) and ATP (A1852, Sigma-Alrich) were dissolved in dH_2_0 and diluted to final concentration in Krebs’. Vehicle was less than 0.1% in the final perfusing solution.

## Results

### Targeted irradiation altered urination patterns

The impact of image-guided bladder irradiation on voiding patterns was assessed through VSA over a range of timepoints: pre-IRR, 2W-IRR, 1M-IRR, 2M-IRR, 3M-IRR, 4M-IRR and 6M-IRR. Post-IRR, the number of void spot locations changed (Fig. 2A,B) with a higher percentage of mice exhibiting more locations at 2W-IRR (46%), 1M-IRR (65%) and 2M-IRR (55%) *vs.* those showing no change, or decreased number of locations. From 3M-IRR, a higher percentage of mice had no change *vs.* those showing increased or decreased number of voiding locations. Consistently, voiding locations occurred away from the edges of the paper, contrary to our expectations from the VSA literature ^24^. This may have been due to the size of the rodent metabolic cages used (approx. 28.5 cm × 46 cm) and/or the 16-h, overnight VSA period that included the awake, dark cycle compared with the shorter recording periods often reported (2–4 h). Void spot counts were measured for each animal across the timepoints (Fig. 2C). Comparison of matched pre- and 2W-IRR data showed increased spot counts (N = 26, *P* < 0.01, Fig. 2D). Further analysis at 2W-IRR showed that mean spot volume decreased (N = 26, *p* < 0.05) and maximum spot volume decreased (N = 26, *P* < 0.01, Fig. 2E,F). Data binning into small (< 50 μL), medium (50–100 μL) and large (> 100 μL) categories showed that a higher percentage of mice had small-volume spots post-IRR (46% vs 19%), and a smaller percentage had large-volume spots post-IRR (19% vs. 31%) (Fig. 2G,H).

### Irradiation reduced neurogenic contraction amplitude

**Fig. 3:**
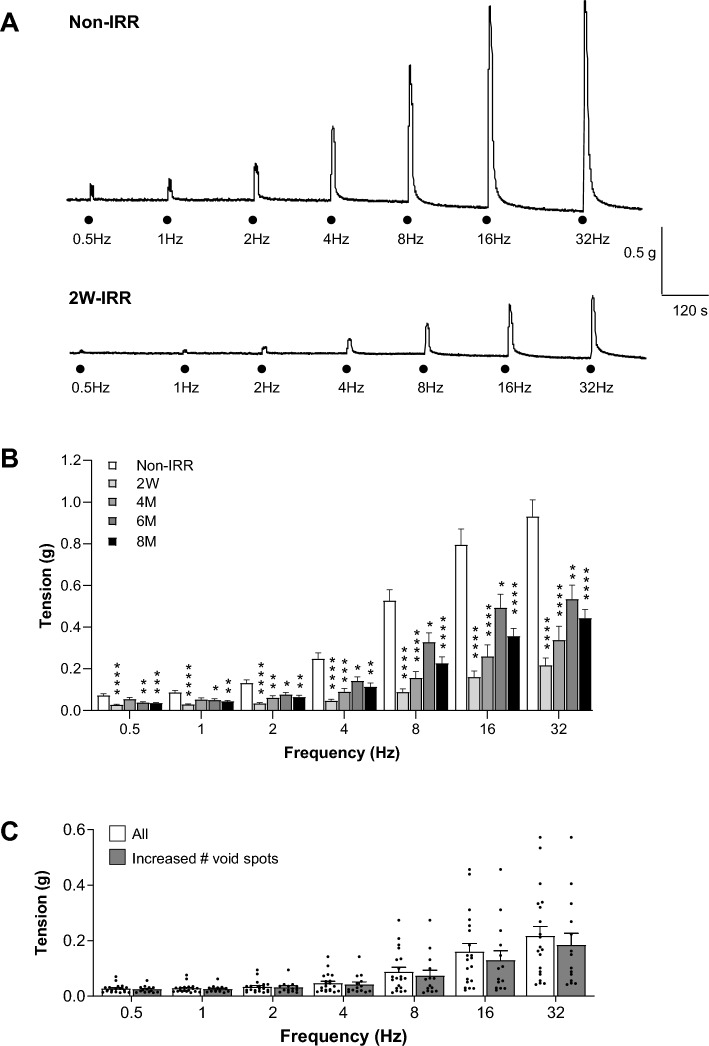
Neurogenic-contractions in bladder strips were reduced post-IRR. **A** Example traces of in vitro tension recordings from tissue strips from a non-IRR and a 2W-IRR mouse bladder. Neurogenic-contractions were evoked by electrical field stimulation (EFS) across a range of frequencies (0.5–32 Hz). **B** Mean contraction amplitude (tension) across the frequency range, for each timepoint (non-IRR, 2W-IRR, 4M-IRR, 6M-IRR and 8M-IRR where M denotes Month) are presented as mean (SEM). At each frequency, contraction amplitude from non-IRR tissues (n = 25, N = 8) was compared with the corresponding data at the 4 post-IRR timepoints: 2W (n = 20, N = 10); 4 M (n = 11, N = 6); 6 M (n = 22, N = 6) and 8 M (n = 39, N = 11) where n and N denote number of tissues and mice respectively. Post-IRR contractions across the timepoints were significantly smaller than non-IRR at the majority of frequencies tested. Data sets were compared with two-way ANOVA and Dunnetts multiple comparison tests. *, **, *** and ****denote *P* < 0.05, *P* < 0.01, *P* < 0.001 and *P* < 0.0001 respectively. An alternative version of this graph comprising granular data points with mean (SD) for Fig. 3B is presented in Supplementary data (Figure S1). **C** As the mean number of voids was significantly increased at 2W-IRR (Fig. 2), neurogenic contraction data for this cohort was disaggregated for data from animals which exhibited increased number of void spots (n = 14, N = 7) to be compared with the whole 2W-IRR cohort (n = 20, N = 10) that were used for in vitro tension recordings. Contraction amplitudes were similar at each frequency tested (*P* > 0.05) whether or not the animals exhibited a changed voiding pattern (mean (SEM)), two-way ANOVA, Bonferroni’s multiple comparison test.

Contractions evoked by electrical field stimulation (EFS) were tetrodotoxin-sensitive (0.1 μM), confirming their neurogenic origin. Representative traces from non-IRR and 2W-IRR bladder strips are shown (Fig. 3A**)**. Mean contraction amplitude was smaller in 2W-IRR (n = 20, N = 10); 4M-IRR (n = 11, N = 6); 6M-IRR (n = 22, N = 6) and 8M-IRR (n = 39, N = 11) than non-IRR strips (n = 25, N = 8) (Fig. 3B, Figure S1). The largest effect occurred at 2W-IRR with some apparent recovery at 4M-IRR, 6M-IRR and 8M-IRR although this did not reach non-IRR levels. A separate series of experiments comparing time-matched 8M-IRR and non-IRR 8M controls also showed reduced neurogenic-contractions in the 8M-IRR group (Figure S2). As the number of void spots was significantly increased at 2W-IRR (Fig. 2), neurogenic-contraction data from animals exhibiting increased number of void spots (n = 14, N = 7 animals) was compared with the whole cohort (n = 20, N = 10). Contraction amplitudes were similar at each frequency in the two groups (*P* > 0.05) (Fig. 3C).

### Irradiation had little impact on cholinergic-contractions or depolarization-contractions but reduced purinergic-contractions

**Fig. 4:**
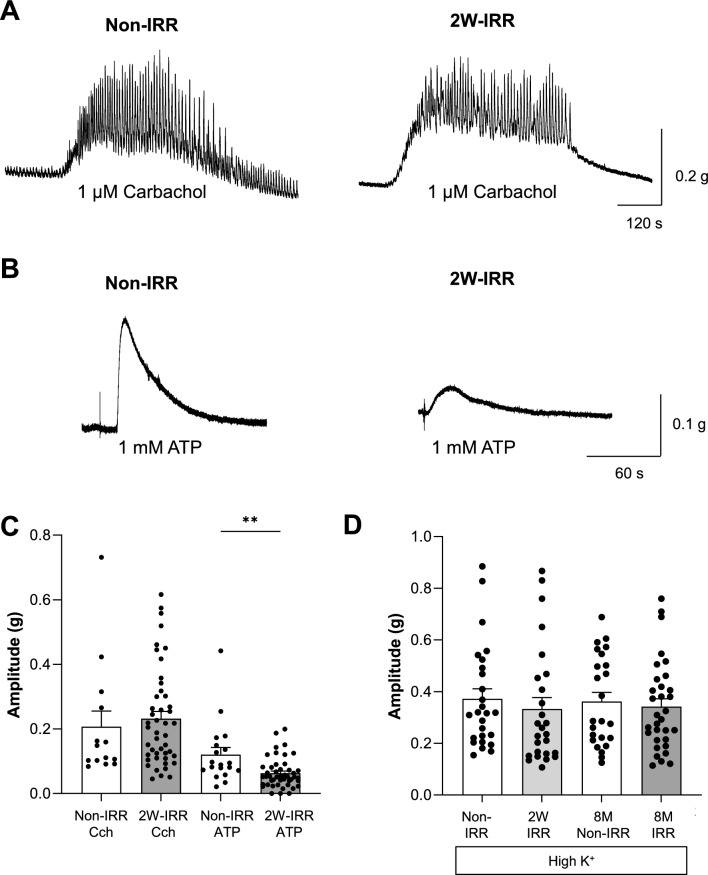
The effect of irradiation on cholinergic, purinergic and receptor-independent contractions in bladder strips. **A** Examples of cholinergic contractions evoked by the muscarinic agonist, carbachol (1 μM) in non-IRR and 2W-IRR bladder strips. These comprised transient contractions superimposed on increased baseline tone. **B** Examples of purinergic contractions evoked by ATP (1 mM) in non-IRR and 2W-IRR bladder strips which comprised a single transient contraction. **C** Summary graph (mean (SEM)) and granular data showing that cholinergic contractions were similar in non-IRR and 2W-IRR tissues (n = 14, N = 10 and n = 46, N = 18 respectively, *P* > 0.05, unpaired t-test). Purinergic-mediated contractions were significantly smaller in 2W-IRR tissues (n = 19, N = 10 non-IRR and n = 46, N = 18, 2W-IRR, *P* < 0.01, unpaired t-test). **D** Receptor-independent contractions, evoked by depolarization with high-K^+^ solution were similar in non-IRR controls (n = 26, N = 8), 2W-IRR (n = 26, N = 8), non-IRR 8M controls (n = 24, N = 7) and 8M-IRR (n = 32, N = 11) (*P* > 0.05) tissues.

Contractility was further assessed by studying receptor-mediated contractions. The muscarinic agonist, carbachol (1 μM) evoked transient contractions superimposed on increased baseline tone (Fig. 4A) that were of similar amplitude and area under curve in non-IRR and 2W-IRR tissues (n = 14, N = 10 and n = 46, N = 18 respectively, *P* > 0.05, Fig. 4C). ATP-evoked purinergic-contractions (1 mM) were smaller in 2W-IRR tissues (Fig. 4B,C, n = 19, N = 10 and n = 46, N = 18 respectively, P < 0.01). Receptor-independent contractions, evoked by depolarization using high-K^+^ were similar in non-IRR (n = 26, N = 8), 2W-IRR (n = 26, N = 8), non-IRR 8M controls (n = 24, N = 7) and 8M-IRR (n = 32, N = 11) (*P* > 0.05, Fig. 4D).

### Potassium channel contribution to contractility was maintained after irradiation

**Fig. 5:**
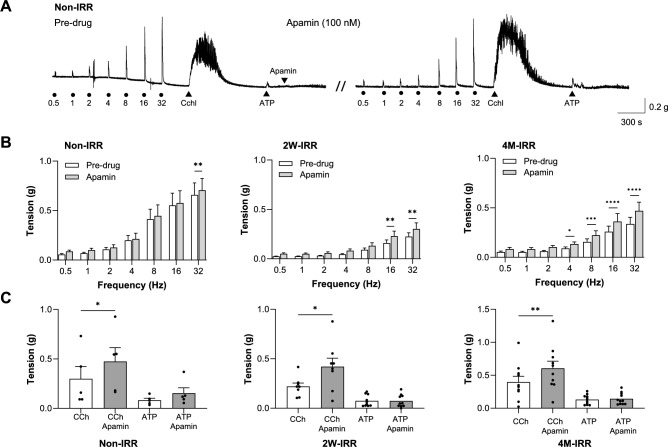
Irradiation had little effect on SK channel contribution to contractility. **A** Typical experiment testing the effect of the SK channel blocker, apamin (100 nM) on neurogenic, cholinergic and purinergic contractions. The trace presented is from a non-IRR bladder; the same protocol was used for post-IRR tissues. Apamin caused a small increase in baseline tension in this recording and also enhanced neurogenic and carbachol-contractions. **B** Summary data showing enhancement of neurogenic-contractions in non-IRR (32 Hz, N = 5, n = 8), 2W-IRR (16–32 Hz, N = 5, n = 10) and 4M-IRR tissues (4-32 Hz, N = 6, n = 11) by apamin (100 nM). Data sets were compared with two-way ANOVA, Sidak’s multiple comparison test with *, **, *** and **** denoting *P* < 0.05, *P* < 0.01, *P* < 0.001 and *P* < 0.0001 respectively. **C** Summary graph (mean (SEM), granular data) for carbachol-contractions and ATP-contractions in the absence and presence of apamin (100 nM) in non-IRR, 2W-IRR and 4M-IRR tissues. Carbachol contractions (N = 5, n = 5; N = 4, n = 8; N = 6, n = 10) were significantly enhanced by apamin (paired t-tests where * and **denote *P* < 0.05 and *P* < 0.01 respectively) at these timepoints, whereas ATP-contractions (N = 5, n = 5; N = 5, n = 10; N = 6, n = 11) were less sensitive to apamin (*P* > 0.05, paired t-tests).

The contribution of small-conductance Ca^2+^-activated K^+^-channels (SK) to contractility was investigated using apamin (100 nM). A typical experiment is presented in Fig. 5A from non-IRR tissue; the same protocol was used for post-IRR strips. Apamin slightly enhanced baseline tension and any low-amplitude spontaneous contractions. Neurogenic-contractions were larger after apamin (32 Hz, non-IRR, 16-32 Hz 2W-IRR, 4-32 Hz 4M-IRR, Fig. 5B, S3). Apamin enhanced carbachol-contractions in non-IRR, 2W-IRR and 4M-IRR strips, whereas ATP-contractions were unaffected (Fig. 5C). These findings suggest that SK contribution to contractility was unaffected by irradiation.

**Fig. 6:**
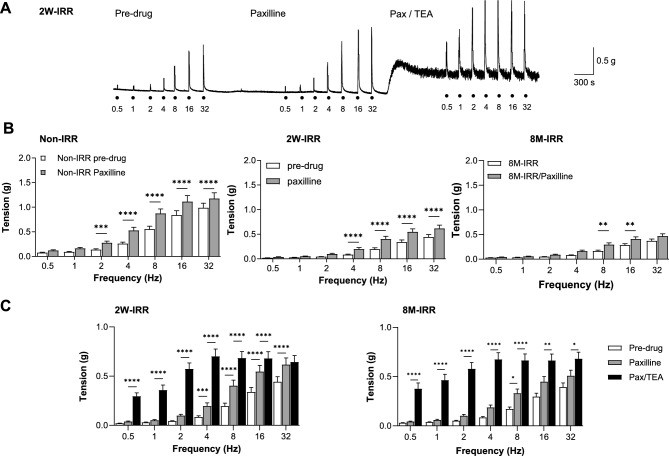
Irradiation had little effect on BK and TEA-sensitive potassium channel contribution to contractility. **A** Example trace of experiment testing the effect of the BK channel blocker, paxilline (1 μM), followed by a combination of paxilline and the pan-K^+^ channel blocker, tetraethylammonium chloride (TEA, 10 mM) on neurogenic-contractions (0.5–32 Hz). The trace presented is from the 2W-IRR cohort. **B** Summary of neurogenic-contractions across the frequency range in the absence and presence of paxilline for non-IRR (N = 5, n = 19), 2W-IRR (N = 8, n = 24) and 8M-IRR (N = 8, n = 28) timepoints. Data sets were compared using 2-way ANOVA and Sidak’s multiple comparison tests. **C** Summary of the effects of paxilline on neurogenic-contractions, followed by a combination of paxilline/TEA at acute 2W-IRR (N = 8, n = 24) and chronic 8M-IRR (N = 6, n = 20) timepoints. Data sets were compared with two-way ANOVA and Sidak’s multiple comparison tests. *, **, *** and ****denote *P* < 0.05, *P* < 0.01, *P* < 0.001 and *P* < 0.0001 respectively. Alternative versions of the plots showing mean, SD and granular points are presented in the Supplementary Data (Figure S4).

The contribution of large-conductance Ca^2+^-activated K^+^-channels (BK) was tested using paxilline (1 μM). An example trace from 2W-IRR (Fig. 6A) shows increased neurogenic-contractions after paxilline, further enhanced by subsequent addition of TEA (pan-K^+^ channel blocker, 10 mM). Paxilline enhanced neurogenic-contractions in non-IRR, 2W-IRR and 8 M-IRR tissues (Fig. 6B). The combination of paxilline and TEA evoked a marked increase in baseline tension and further increased neurogenic-contractions at acute 2W-IRR and chronic 8M-IRR timepoints (Fig. 6A,C). Plots of paxilline-sensitive and Pax/TEA-sensitive contractions show similar patterns (Figure S4). These experiments demonstrate the functional relevance of BK and other K^+^-channels to the maintenance of baseline tension and their ability to act as a ‘brake’, limiting the amplitude of neurogenic-contractions. As these properties were maintained in post-IRR tissues, it is likely that BK and other K^+^ channel activity persisted.

## Discussion

This study demonstrated that image-guided bladder irradiation with a single fraction of 20 Gy, evoked radiation-bladder toxicity in female mice, manifested as altered voiding patterns and diminished neurogenic-contractions. We discovered that at 2W-IRR, almost half of the mice had increased voiding locations and increased numbers of void spots, with the majority exhibiting smaller mean- and maximal void spot volumes. Furthermore, neurogenic-contractions of bladder strips were smaller than non-IRR. Neurogenic-contraction amplitudes were larger at later timepoints; however, they remained smaller than non-IRR. Depolarization-contractions were unaffected showing that bladder contraction per se was unaffected by irradiation. Carbachol-contractions were similar; however, ATP-contractions were smaller post-IRR. The contribution of K^+^ channels to contractions was apparently unchanged after irradiation.

Our finding that a single-fraction of 20 Gy, delivered to the bladder via image-guidance, evoked voiding changes in the acute phase, 2W-IRR, is consistent with studies using other modalities. External-beam, single-dose, 20 Gy to the female rat pelvis caused increased urination frequency and smaller micturition volume (VSA, 2W-IRR) ^26^. Earlier studies with this methodology reported radiation-responding animals as those with < 50% of pre-IRR bladder capacity ^8,11,27^. The present study found that at 2W-IRR, around 50% of the mice had increased voiding spot locations andmore void spots vs. pre-IRR; moreover, the majority had decreased mean- and maximal volume of void spots. These findings are consistent with other image-guided bladder irradiation studies of female mice ^16,17^. In contrast, in male mice, a single-dose of 30 Gy under image-guidance only correlated with increased urinary frequency and reduced volume of individual voids at later timepoints (> 14W-IRR), not at 4W-IRR ^18^. These sex-based differences in rodent bladder voiding after image-guided irradiation are incompletely understood.

Altered urination reported here, and by others might suggest that 2W-IRR cohorts experience urgency, frequency or bladder irritation, commonly reported by patients in the acute phase ^6^. VSA is a commonly-used technique due to convenience and cost-effectiveness ^25^; however, it cannot provide information on the time of voiding nor the inter-micturition interval. This work could be advanced using devices that enable voiding data to be recorded in real-time and for longer durations ^28,29^. Unexpectedly, in the present study, VSA voiding locations typically occurred away from the edge of the paper, in contrast to studies where voids are visible on edge of the paper, representing the cage boundary ^24^. As thigmotaxis (wall-seeking) correlates with increased emotionality or anxiety in mouse behavioural studies ^30^, the VSA conditions used here (mice acclimatisation to the metabolic cages, subsequent overnight 16-h measurements during the typical awake period for mice compared with more commonly used 2–4 h VSA during the day), may have fostered lower-levels of anxiety and therefore, voiding occurred away from the cage boundaries. Nevertheless, the possibility remains that the voiding location pattern observed here may be atypical.

The higher number of small-volume voids at 2W-IRR might be due to impaired bladder contraction and/or defects in the bladder outlet or urethra, resulting in detrusor sphincter dyssynergia. A recent study of pelvic radiation-toxicity in female rats reported damage to internal and external urethral sphincters, exhibited as increased urethral neurogenic-contractions 4W-IRR and a reduced smooth muscle composition ^31^. Alternatively, post-IRR, sensation of bladder fullness might be altered, leading to more frequent voids, potentially due to urothelial thinning and reduced urothelial junctional proteins ^16^ causing a leaky urothelium, irritation of cellular elements in the lamina propria by urine and subsequent aberrant afferent signalling. This has not yet been directly tested.

We previously reported decreased neurogenic-contractions in acutely-irradiated ex vivo guinea-pig bladder strips. This effect was observed in intact, not detrusor (mucosa-free) strips ^19^. Here, we found that neurogenic-contractions in post-IRR (full-thickness) bladder strips were smaller from 2W-IRR onwards. Neurogenic-contractions were reduced, whether or not the animal exhibited altered VSA patterns, suggesting that irradiation has early effects on neurogenic-contractions, and that in around half of the animals, this translated to altered urination. Up to 8M-IRR, neurogenic-contractions increased; however, they did not reach non-IRR amplitudes. Smaller neurogenic-contractions is a consistent post-IRR, pre-clinical finding, whether pelvic radiation was delivered by external-beam and shielding to mice ^20,31^, in directly-irradiated ex vivo guinea-pig tissue ^19^ or here, in in vivo image-guided bladder irradiation. Reduced neurogenic-contractions might explain increased voiding and reduced void (spot) volume due to voiding contractions being less efficient. In addition, if the urethra was affected with increased post-IRR urethral contractions ^31^, this might act against the compromised bladder neurogenic-contraction and together result in voiding dysfunction.

Irradiation did not affect the ability of bladder strips to contract per se, consistent with acutely-irradiated guinea-pig bladder tissue ^19^ and in vivo irradiated rat pelvis ^20^, confirming that smooth muscle contraction mechanisms were largely unaffected. Receptor-mediated contractions in mouse bladder e.g. mediated by M3 (muscarinic) or P2X1 (purinergic) receptors may be sensitive to irradiation and it was found in the present study that ATP-contractions were smaller post-IRR whereas carbachol-contractions were unchanged. This former is consistent with reports for rat bladder where contractions evoked by the P2X stable agonist, α,β-methylene-ATP, were smaller in post-IRR tissue ^20^. Investigation of the function and/or expression of purinergic receptors should be investigated in the post-IRR mouse bladder; of note in rat bladder, protein expression of P2X1 was unchanged post-IRR ^20^. The cholinergic and purinergic mechanisms underpinning neurogenic contractions which we previously investigated in acutely-irradiated guinea-pig bladder tissue ^19^ and others investigated in rat bladder ^20^ were not dissected in the present study. Further study of pre- and post-synaptic mechanisms are required to understand the smaller neurogenic-contractions in the post-IRR bladder, which in normal mouse bladder are co-mediated by muscarinic and purinergic pathways ^32,33^.

Bladder contractility is modulated by K^+^-channel activity, acting as a brake, dampening contractions, particularly during bladder filling. We examined the contribution of K^+^-channels to neurogenic and receptor-contractions using pharmacological inhibitors. Neurogenic-contractions were enhanced by apamin in both non-IRR and post-IRR tissues, but to a lesser extent than reported for normal mouse bladder by others who used higher concentrations (100 nM present study, 230 nM by ^34^ or 1 μM by ^35^). Furthermore, apamin enhanced carbachol-contractions (but not ATP-contractions) in non-IRR and post-IRR bladder. A similar effect of apamin on carbachol-contractions in normal mouse bladder has been reported by others ^36^. Inhibition of BK and TEA-sensitive K^+^-channels had comparable effects on neurogenic-contractions in non-IRR and post-IRR tissues. This data confirms that functional expression of SK, BK and other K^+^ channels is unaffected by the irradiation conditions used here, and that they continue to modulate bladder contractility.

A strength of this study was the use of image-guidance to target the bladder with high precision and dosimetry certainty whilst also evaluating voiding patterns and bladder contractility. The mice tolerated the radiation protocol and therefore the effects on bladder physiology could be examined in the context of limited systemic pathophysiology. The single-dose given is consistent with the pre-clinical radiation research field and aligns with hypofractionated treatments emerging clinically in prostate cancer treatment regimens comprising fewer fractions of larger doses ^37^. The use of young adult, female mice in the present study which correlates with cervical cancer cohorts, may limit translation of the findings to prostate cancer or bladder cancer treatment and this would be better modelled with older mice and inclusion of male cohorts. Further pre-clinical investigation to address limitations of the study should incorporate image-guided, targeted irradiation protocols, for a range of doses, fractionation schedules and timepoints. This should include physiological mechanistic studies on the bladder and urethra of both biological sexes, with young adult and older adult models, to elucidate why altered neurogenic-contractions occur post-IRR in both areas of the lower urinary tract. Further research will therefore inform our understanding of medium-term urinary toxicity and illuminate preventative and/or treatment measures.

## Conclusions

In conclusion, in vivo image-guided, targeted bladder irradiation with a single fraction (20 Gy) induced altered urination patterns in the acute phase, coincident with impaired neurogenic-contractions which might contribute to incomplete voiding. Post-synaptic muscarinic mechanisms were apparently unaffected, whereas purinergic contractions were reduced. While the underlying mechanisms are incompletely understood, the ability of the bladder smooth muscle to contract was not affected, neither was K^+^-channel modulation of bladder contractility compromised. The study supports use of advanced preclinical approaches, in the investigation of radiation-bladder toxicity to enable the underlying pathophysiology to be further investigated.

## Supplementary Information

Supplementary Information.

## Data Availability

The data that support the findings of this study are available from the corresponding author upon reasonable request.
