# Supplementary material for: Investigation of image-guided in vivo irradiation on voiding patterns and bladder contractility in female mice
Source: Sci Rep. 2025 Dec 13;16:518. doi: 10.1038/s41598-025-30020-6 (PMC12775023; doi:10.1038/s41598-025-30020-6)
Supplement: Supplementary file 1 — Supplementary Information. [file 41598_2025_30020_MOESM1_ESM.docx]

**Supplementary Information**


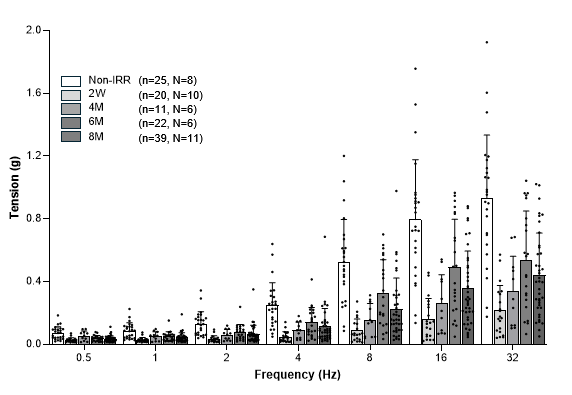


**Figure S1. Neurogenic-contractions in bladder strips were reduced post-IRR**

Alternative version of **Figure 3B.** Mean contraction amplitude across the frequency range, for each timepoint (non-IRR, 2W-IRR, 4M-IRR, 6M-IRR and 8M-IRR where M denotes Month) are presented as mean (SD) with granular points. At each frequency, contraction amplitude from non-IRR tissues (n=25, N=8) was compared with the corresponding data at the 4 post-IRR timepoints: 2W (n=20, N=10); 4M (n=11, N=6); 6M (n=22, N=6) and 8M (n=39, N=11) where n and N denote number of tissues and mice respectively. Statistical tests are presented in the version of **Figure 3B**


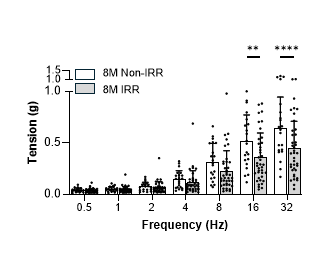


**Figure S2. Neurogenic-contractions 8M-IRR compared with time-matched controls**

Summary of mean contraction amplitude across the frequency range for 8M-non-IRR controls (N=6, n=21) and 8M-IRR cohorts (N=11, n=39). Similar to the data in **Figure 3B** and **Figure S1**, neurogenic-contractions in 8M-IRR were smaller across the frequency range with statistically significant decreases at 16Hz and 32Hz (two-way ANOVA with Bonferroni’s multiple comparison tests, ** and **** denotes *P*<0.01 and *P*<0.0001 respectively).


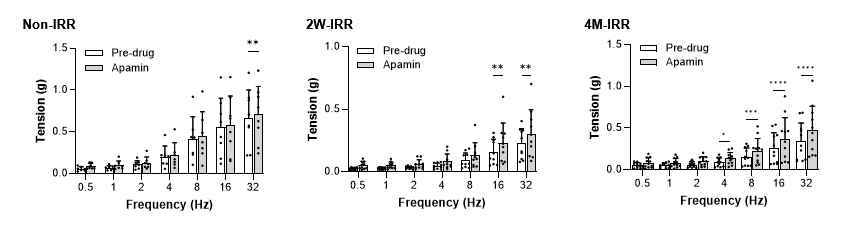


**Figure S3. Irradiation had little effect on SK channel contribution to neurogenic-contractions**

Alternative version of **Figure 5B.** Summary data (mean and SD with granular data) showing enhancement of neurogenic-contractions in non-IRR (32 Hz, N=5, n=8), 2W-IRR (16-32 Hz, N=5, n=10) and 4M-IRR tissues (4-32Hz, N=6, n=11) by apamin (100 nM). Data sets were compared with two-way ANOVA, Šídák's multiple comparisons test with *, **, *** and **** denoting *P*<0.05, *P*<0.01, *P*<0.001 and *P*<0.0001 respectively.


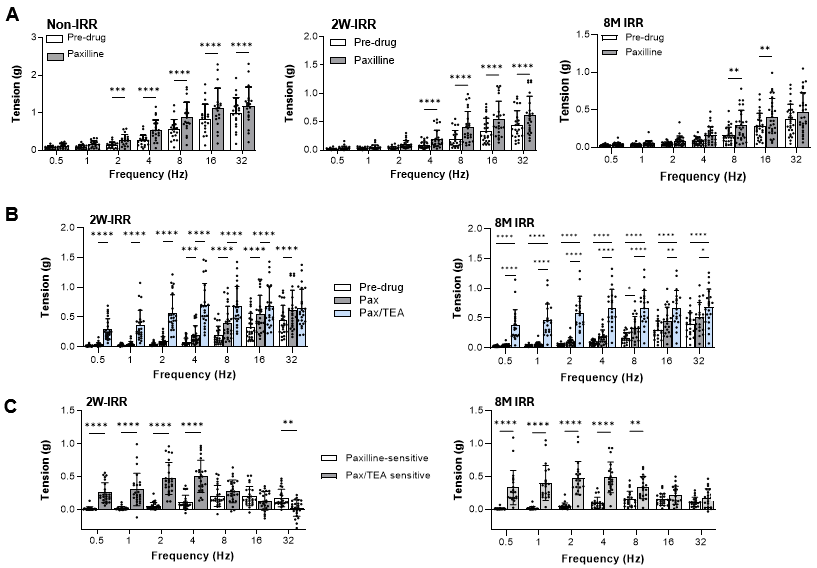


**Figure S4. Irradiation had little effect on BK and TEA-sensitive potassium channel contribution to contractility**

Alternative versions of the plots in **Figure 6B and C** showing mean, SD and granular points.

**A.** Summary of neurogenic-contractions in the absence and presence of paxilline for non-IRR (N=5, n=19), 2W-IRR (N=8, n=24) and 8M-IRR (N=8, n=28) timepoints. Data sets were compared using 2-way ANOVA and Šídák's multiple comparisons test.

**B.** Summary of the effects of paxilline on neurogenic-contractions, followed by a combination of paxilline/TEA at acute 2W-IRR (N=8, n=24) and chronic 8M-IRR (N=6, n=20) timepoints. Data sets were compared with two-way ANOVA and Šídák's multiple comparison tests.

**C.** Summary showing paxilline-sensitive and paxilline/TEA-sensitive components of neurogenic-contractions at 2W-IRR and 8M-IRR timepoints. Data sets were compared with two-way ANOVA and Šídák's multiple comparison tests.

*, **, *** and **** denote *P*<0.05, *P*<0.01, *P*<0.001 and *P*<0.0001 respectively.
